# Is there a relationship between fetal sex and placental pathological characteristics in twin gestations?

**DOI:** 10.1186/s12884-018-1896-9

**Published:** 2018-07-04

**Authors:** Shayesteh Jahanfar, Kenneth Lim

**Affiliations:** 1 School of Health Sciences Building 2242, Central Michigan University, Mount Pleasant, MI 48859 USA; 2 Division of Maternal Fetal Medicine, BC Women’s Hospital, Vancouver, Canada

**Keywords:** Placenta, Cord, Pathology, Twin pregnancy

## Abstract

**Background:**

Placenta plays a central role in mediating growth and development of fetuses. Sex-specific placentas may complicate this role.

**Methods:**

The study aimed at investigating the association between fetal sex and placental pathological findings in twin gestations using generalized estimating equation modeling. We used a large population-based clinical data born in British Columbia (BC) and linked the fetal-maternal data to hand-searched pathology reports of 1493 twin placentas from twins born in BC Women hospital. We analyzed the data using generalized estimating equations taking the cluster nature of twins into consideration.

**Results:**

About 26.5% of twins were monochorionic and 73.5% were dizygotic. Most twins were male (51.3%). About 2/3 of twins were sex concordant (66.6%). Of the sex concordant twins, similar percentages were male-male (34.7%) and female-female (31.2%). Of the sex discordant twins, the male-female (33.3%) group constituted about 1/3 of the whole population.

Adjusted for chorionicity, birth weight discordance ≥30% and gestational age, the odds of chorionitis (1.38, 95% CI = 1.04–1.84), anastomosis (1.63, 95% CI = 1.22–2.19), unequal sharing of placenta (1.72, 95% CI = 1.11–2.64), placental inflammation (1.30, 95% CI = 1.05–1.62) and lesions (1.83, 95% CI = 1.02–3.31) were higher in male twins compared with females. Twins of either sex from sex-discordant pairs were less likely to have placental anastomosis compared to the reference category. Males from male-male pairs had higher odds of unequal placental sharing (74% higher) and composite inflammation (52% higher) compared with the reference twins.

**Conclusion:**

Our findings suggest a relationship between sex and placental pathological results.

## Background

Adverse perinatal outcomes and fetal growth have been found to be associated with fetal sex [1]. The placenta, as an important part of the fetus’s nutritional route, plays a central role in transporting amino acids hence mediating growth and development of fetuses [2, 3]. Sex differences in growth discordance and fetal/neonatal morbidities and mortality are likely to be mediated by sex-specific placenta functions. None of the findings in the literature has led clinicians to pay attention to sex during perinatal screening, mostly because the mechanisms that confer these differences between the sexes are unknown. It is also possible that the association between adverse perinatal outcomes and fetal sex is not that strong or that other factors, rather than fetal sex, such as chorionicity play a stronger role.

**Fig. 1:**
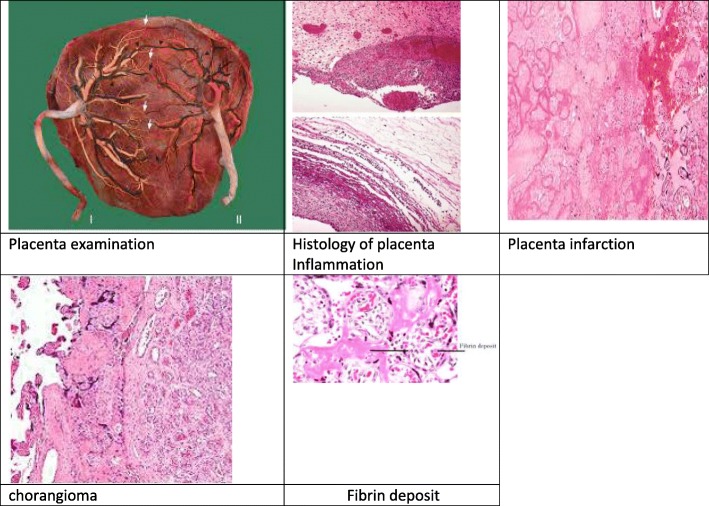
Images of placenta under study parameters

A paucity of data exists in the literature regarding the relationship between sex and pathology of the placenta and cord. Previous studies of pathology findings tend to focus on factors other than infant sex, such as birth weight discordance (BWD) [4]. If sex was noted as a secondary risk factor of interest, then simple analytical differences between male and female in the placenta’s adverse outcomes were investigated rather than the risk of adverse pathological events by sex pairing [5]. For example, in singleton pregnancies, intra-uterine infection/inflammation of male fetuses has been attributed to higher mortality of male newborns. A histological examination of placentas and umbilical cords of 446 infants born at 23 to 32 weeks, cultured for aerobic and anaerobic bacteria, showed that male infants were significantly more likely to have placental membrane bacterial infection than female infants [6]. Figure 1 shows images of placenta histology and various complications. Literature has not much to offer about twin data. Our study aimed to investigate several pathological characteristics of the placenta associated with fetal sex.

## Methods

A retrospective cohort of twins born in British Columbia Children and Women’s (C&W) Hospital for a period of a decade (2000–2010) was studied. Data were abstracted from pathology reports by single reviewer systematically and linked with information from mothers and babies’ suites. The pathology data were then linked to delivery outcome data such as gestational age and birth weight by Perinatal Services British Columbia (PSBC) [7]. The link was possible using the personal health number, maternal and baby identification number and date of birth. The final linked records were stored on Secure Research Environment on a Virtual Private Network.

Sex pairing, an exposure variable, was a composite of the twin’s sex and with what type of twin pair he or she was identified: male from a male-male pair, male from a male-female pair, female from a female-male pair, and female from a female-female pair. Infants from a female-female pairing were considered as the referent group. All placenta and cord pathology data were outcome variables.

The pathology data of placenta were collected and labeled as A (1 cord clamp) or B (2 cord clamps) according to birth order. Placentas were placed in plastic bags after delivery and kept at 4 degrees centigrade until processed, usually within 24 h of delivery. Placenta examination was carried out in the pathology department of the C&W hospital in BC, Canada. Pathologists who examined the placentas had access to the clinical information. The placentas were placed on a clean surface, adherent clots were removed, and the membranes and umbilical cords were excised before they were weighed. There was a systematic approach to attribute placental mass to each twin so that the total placental weight was recorded for each placenta. In DC placentas with fused placentas, the proportion of placenta belonging to each twin was determined by measuring the length, width, and thickness in each of the two placental disks. Measurement of placental thickness was carried out in three areas of the placental disk, and the mean thickness was then recorded.

Cord length and its distances from the placenta margin, from the membrane and from the other cord were also measured. Umbilical cord insertions into the disc of the placenta and more than 1 cm away from the marginal border were defined as (para) central, cord insertions within 1 cm of the disc edge were defined as marginal and cord insertions directly into the membranes were defined as velamentous. The number of vessels in the cord was recorded. A composite variable was created from cord properties inclusive of cord prolapse, number of cord vessels less than 3, and existence of cords knots or entanglements.

The evaluation of placental chorionicity was performed by examination of the inter-twin membrane. Separated twin placentas were examined in the same way as those of singletons. Fused placentas can be MC or DC. The dividing membrane was examined to identify chorionicity. The dividing membrane in a MC pregnancy is thin and translucent without any chorionic layer, while that of a DC placenta is thicker as it contains two chorionic layers between the amniotic sacs. The dividing membrane was then sampled as a membrane roll or in “T section” form. Identification of T form was considered confirmation of chorionicity.

Equal placental sharing was defined as 40 to 60% of the placenta attributed to each twin. We chose this range because preliminary data revealed that twins with 40/60 sharing and 50/50 sharing had similar gestational ages at delivery and degree of BWD [8]. Unequal placental sharing was defined as 1 twin receiving blood from more than 60% of the placenta.

In histological examination of the placenta, vascular-thrombotic lesions (infarction, chorangioma, subchorial fibrin deposition, and retro placental hematoma) were recorded (Image 1). Arteries were identified as vessels that are situated superficial in relation to the veins. A composite score was created for all of the pathological lesions for the purpose of analysis.

Anastomosis between fetal vessels was recorded. Anastomosis was identified by the presence of an impaired vessel from one twin feeding an area drained by the co-twin. Injection studies were performed in fresh specimen to identify unidirectional arteriovenous shunt(s) between donor and recipient.

Placentas were also assessed for maternal or fetal inflammatory response corresponding to chorionisits, chorioamnioitis, and chorionic villi inflammation. A composite variable was created using these items plus cases with inflammation or infection of the maternal or fetal side of the chorionic membrane.

Diagnosis of twin to twin transfusion syndrome (TTTs) was made by the referring obstetrician and was designated on the pathology requisition. Placenta abruptio and invasive of trophoblast such as placenta accreta was also recorded.

Completed pathology reports for twins were printed from online pdf records or from hard copies of pathology records stored in hospital charts. Data were then abstracted from these records into an Excel database. Twin pregnancies were included in the data if mothers and babies were linked, babies had a calculated estimated gestational age at birth ≥20 weeks, and the mother did not have a termination procedure.

Twins with gestational age at birth ≥20 weeks, were included. We excluded cases with congenital anomalies, TTTs, < 500 g’ birth weight, one stillbirth, those who had a reduction procedure in a multiple pregnancy greater than two (3 to 2 or 4 to 2), and papyrus placentas. We chose to exclude the latter category of vanishing twins from this analysis because of an association between sex discordance and vanishing twins [9].

Incidences of placenta and cord characteristics were compared between male and female.

Twins are of special interest because they provide naturally matched pairs where the confounding effects of many potentially causal factors (such as maternal nutrition or gestation length) may be removed by comparisons between twins who share them. Epidemiological ‘risk factor analysis’ uses the regression model, but it is not straightforward to transfer standard regression methods to twin data, because the analysis needs to reflect the paired structure of the data, which induces correlation between twins [10]. Bivariate analysis was used to determine significant variables that were to be included in the generalized estimating equation (GEE) regression analysis. We used this method of analysis because we were mindful of the correlation between twins and the cluster nature of twins in to account. We used GEE analysis throughout the whole paper. However, we choose two approaches: 1. “Population average approach” to analyze the relationship between sex (all males compared with all females given the pair nature of twins, this comparison takes place while linking twin 1 with twin 2) and pathological findings. 2. “Subject-specific approach” or “pair-specific approach” to estimate the total association of fetal sex pairing (female-female [FF] versus male-male [MM] versus male-female [MF] where twin 1 and twin 2 are linked in each group of comparison) on pathological outcomes. By comparing these two approaches, we can present a complete picture for the impact of sex on pathological findings. Therefore, we investigated the relationship between sex pairing and pathological findings.

## Results

There were 1493 pairs (*n* = 2896) in the analytical data, from which 26.5% (*n* = 768) had monochorionic (MC) and 73.5% (*n* = 2128) had dichorionic (DC) placentas. Most twins were male (51.3%). About 2/3 of twins were sex concordant (66.6%). Of the sex concordant twins, similar percentages were MM = 34.7% and FF = 31.2%. Of the sex discordant twins, the MF = 33.3% group constituted about 1/3 of the whole population. Female-female group was considered the reference group for further analysis.

MC twins were on average 341 g lighter than their DC (MC birth weight = 1991.21 ± 761.95 versus DC birth weight = 2332.03 ± 646.68, *p* = 0.01) counterparts and were delivered at an earlier mean gestational age (MC: 33.02 ± 4.30 versus DC: 34.69 ± 3.32, *p* = 0.01). The mode of delivery was more frequently cesarean section than vaginal delivery (62.2% versus 37.8%, respectively). DC twins a had higher frequency of cesarean section than MC twins (*p* = 0.01).

### Relationship between pathology results and fetal sex: A population average approach

The sample was composed of 1534 males (51.4%) and 1452 females (48.6%). Two cases were identified with unknown sex and were excluded from the analysis.

Higher frequencies of chorionitis, anastomosis, unequal placenta sharing, inflammation and placenta lesions were found in males compared with female twins. Cord length was about 1.5 cm longer in males compared with female twins. These variables were then further analyzed by regression analysis (Table 1).

**Table 1:** Characteristics of placenta and cord, overall and stratified by sex for twins born in C&W hospital (1493 pairs, *n* = 2986)

	Overall	Male	Female	P
		*N* = 1534	*N* = 1452	
Placenta				
Chorionic villi inflammation	264 (9.2%)	149 (10.1%)	115 (8.3%)	0.09
Chorionitis	208 (7.3%)	121 (8.2%)	87 (6.3%)	0.04
Anastomosis	252 (8.8%)	155 (10.6%)	96 (6.9%)	0.01
Unequal placenta sharing^a^	142 (32.6%)	84 (37.2%)	58 (27.6%)	0.04
Composite of inflammation	378 (13.2%)	214 (14.6%)	164 (11.8%)	0.03
Composite placenta lesions	50 (1.7%)	33 (2.2%)	17 (1.2%)	0.05
Cord composite	9 (0.3%)	6 (0.4%)	< 5(< 0.3%)	0.36
Placenta others	36 (1.3%)	20 (1.4%)	16 (1.2%)	0.74
Placenta weight, g, mean ± SD	532.62 ± 228.97	536.57 ± 228.44	528.56 ± 229.68	0.46
Placenta length, cm, mean ± SD	22.02 ± 5.14	22.16 ± 5.21	21.87 ± 5.06	0.23
Placenta width, cm, mean ± SD	532.62 ± 228.97	17.05 ± 16.94	16.94 ± 3.93	0.55
Placenta thickness, cm, mean ± SD	2.12 ± 0.47	2.11 ± 0.51	2.13 ± 0.41	0.37
Cord				
Cord length, cm^a^, mean ± SD	27.33 ± 12.70	28.03 ± 13.14	26.57 ± 12.21	0.02
Cord distance from margin, cm, mean ± SD	4.60 ± 2.27	4.58 ± 2.23	4.63 ± 2.31	0.59
Cord distance from membrane, cm, mean ± SD	6.52 ± 5.14	6.63 ± 3.96	6.40 ± 4.01	0.41
Cord distance from other cord, cm, mean ± SD	14.14 ± 8.43	6.63 ± 3.95	6.40 ± 4.01	0.24
Cord insertion type				
Marginal	1981 (69.3%)	1029 (78.0%)	950 (76.2%)	0.41
Central	469 (16.4%)	228 (17.3%)	241 (19.3%)	
Velamentous	118 (4.1%)	62 (4.7%)	56 (4.5%)	

^a^Mann Whitney test

*SD* standard deviation

Unequal placental sharing was defined as 1 twin receiving blood from more than 60% of the placenta

Composite of inflammation: Chorioamnioitis, chorionic villi inflammation, chorionitis, membrane inflammation and other abnormalities of amnion and chorion

Composite placenta lesions: Placenta infarction, placenta abnormalities, chorangioma, hematoma and placenta morphological and functional anomalies

Cord composite: Number of cord vessels less than 3, compression of umbilical cord problems, and other unspecified conditions related to cord

Placenta others: Placenta previa, placenta abruptio, placenta accreta, and other types of placenta separations that causes hemorrhage

Compared with females, the odds of chorionitis, anastomosis, unequal sharing of placenta, placental inflammation and lesions were higher in male twins, adjusted for chorionicity, BWD ≥ 30% and gestational age (Table 2).

**Table 2:** Regression analyses of pathology findings, comparing male and female twins born in C&W hospital (1493 pairs, *n* = 2986)

	Unadjusted (95% CI)	Adjusted (95% CI)
Chorionitis	1.34 (1.01–1.79)	1.38 (1.04–1.84)^a^
Anastomosis	1.59 (1.22–2.07)	1.63 (1.22–2.19)^b^
Unequal placenta sharing	1.5 (1.03–2.33)	1.72 (1.11–2.64)^a^
Composite of inflammation	1.27 (1.02–1.58)	1.30 (1.05–1.62)^a^
Composite placenta lesions	1.86 (1.03–3.35)	1.83 (1.02–3.31)^a^
Cord length	1.46 (0.48–2.44)	1.35 (0.39–2.30)^c^

Ref: Female; ^a^Adjusted for chorionicity

BMI ≥ 30% and gestational age

^b^Adjusted for chorionicity and gestational age

^c^Adjusted for BMI ≥ 30% and gestational age

&Two individual twins with unknown sex were excluded

Unequal placental sharing was defined as 1 twin receiving blood from more than 60% of the placenta

Composite of inflammation: Chorioamnioitis, chorionic villi inflammation, chorionitis, membrane inflammation and other abnormalities of amnion and chorion

Composite placenta lesions: Placenta infarction, placenta abnormalities, chorangioma, hematoma and placenta morphological and functional anomalies

### Relationship between pathology results and fetal sex: A subject-specific approach

We were interested in estimating the total association of fetal sex pairing on pathology adverse outcomes. Of the eight categorical outcomes under study, unequally shared placenta and anastomosis and placental inflammation were the most common (Table 3). The frequency of seven variables (anastomosis, unequal placenta sharing, composite of inflammation, placenta weight, length and width. Cord length was also significantly different between sex pairs of different types) among sex groups is significate different as the *P* value was found to be less than 0.05 in each comparison. Cord length was also significantly different between sex pairs of different types. However, except for anastomosis and unequal placenta sharing, the difference between categories was clinically small. Thus, no further analysis was performed.

**Table 3:** Comparison between frequency of placental /cord pathology findings in sex pairing groups in terms of twins born at C&W hospital (1493 pairs, *n* = 2986)

	F from FF	M from MM	M from MF	F from MF	P
	*n* = 925	*n* = 1005	*n* = 483	*n* = 483	
Placenta					
Chorionic villi inflammation	74 (8.0%)	112 (11.1%)	37 (8.0%)	41 (8.8%)	0.07
Chorionitis	53 (5.7%)	89 (8.9%)	32 (6.9%)	34 (7.3%)	0.07
Anastomosis	88 (9.5%)	144 (14.3%)	11 (2.4%)	8 (1.7%)	0.01
Unequal placenta sharing	47 (30.1%)	72 (42.1%)	12 (21.8%)	11 (20.4%)	0.01
Composite of inflammation	104 (11.2%)	160 (15.9%)	54 (11.6%)	60 (12.9%)	0.01
Composite placenta lesions	10 (1.1%)	23 (2.3%)	10 (2.2%)	7 (1.5%)	0.19
Cord composite	< 5(< 0.5%)	5 (0.5%)	< 5(< 1.0%)	0 (0.0%)	–
Placenta others	11 (1.2%)	17 (1.7%)	< 5(< 1.0%)	5 (1.1%)	0.38
Placenta weight, g, mean ± SD	557.17 ± 231.99	558.17 ± 232.75	495.46 ± 214.42	475.52 ± 215.88	0.01
Placenta length, cm, mean ± SD	22.17 ± 4.91	22.46 ± 5.24	21.55 ± 5.11	21.30 ± 5.29	0.01
Placenta width, cm, mean ± SD	17.44 ± 3.93	17.47 ± 4.20	16.36 ± 3.79	16.99 ± 4.02	0.01
Placenta thickness, cm, mean ± SD	2.16 ± 0.41	2.11 ± 0.48	2.11 ± 0.54	2.08 ± 0.42	0.10
Cord					
Cord length, cm*, mean ± SD	25.97 ± 11.98	28.08 ± 13.22	27.92 ± 12.87	27.78 ± 12.58	0.01
Cord distance from margin, cm, mean ± SD	4.62 ± 2.39	4.53 ± 2.41	4.67 ± 2.22	4.64 ± 2.15	0.75
Cord distance from membrane, cm, mean ± SD	6.45 ± 4.08	6.62 ± 3.99	6.66 ± 3.89	6.30 ± 3.83	0.85
Cord distance from other cord, cm, mean ± SD	13.35 ± 5.71	14.75 ± 11.52	13.68 ± 5.45	14.73 ± 8.43	0.16
Cord insertion type					
Marginal	612 (75.0%)	724 (79.5%)	305 (74.8%)	338 (78.4%)	0.13
Central	162 (19.9%)	143 (15.7%)	85 (20.8%)	79 (18.3%)	
Velamentous	42 (5.1%)	44 (4.8%)	18 (4.4%)	14 (3.2%)	

Placenta others refers to placenta previa, placenta abruptio, placenta accreta, and other types of placenta separations that causes hemorrhage

Categorical data compared using Chi-square test

Continues variables compared with Anova

*SD* Standard deviation, *M* male, *F* female, *FF* female-female, *MM* male-male, *MF* male-female

Compared to females from the FF group (the reference category), twins of either sex from mixed-sex pairs were less likely to have anastomosis. Males from MM pairs had a statistically significant increase in their odds of anastomosis compared with females from FF pairs (1.71; 95% CI = 1.26–2.33).

The odds of unequal placental sharing were highest in males from male-male pairs (1.75, 95% CI = 1.14–2.69) compared with the females of concordant pairs (FF, data is not shown). After adjustment for chorionicity, the odds remained statistically significantly high (1.74, 95% CI = 1.13–2.69) compared to the reference category. Similarly, the adjusted (for chorionicity and gestational age) odds of composite inflammation were higher in males with male-male status compared to females of female-female pairs (1.52. 95% CI = 1.18–1.94).

Linear regression analyses were used to analyze the association between sex pairing and placenta weight, length, and width. From one level of sex pairing to the other, placenta weight decreases, on average, by about 10 g. In other words, a male from either a MM pair or a mixed-sex pair has a statistically significant reduction in their placental weight compared with infants from a FF twin pair. Like placenta weight, placenta sizes (length and width) on average were shorter among other categories compared with females of FF pairs, the reference category. These values are not clinically significant.

## Discussion

This analysis of a retrospective cohort of pathological data revealed an association between twin sex and pathology characteristics of placenta and cord.

Given that the intra-uterine environment shared by a set of twins could impact the outcomes of interest in this study, we utilize two analytical approaches to investigate the role of sex in relation to pathological outcomes. We first analyzed the data using a population-average approach where a selected twin from the population is compared with another twin who is also selected from the population. We then compared each twin with his or her mate, illustrating the use of a subject-specific-model rather than a population-based approach. Hence, sex pairing comparisons were adopted for analysis.

Based on the population average approach, we found that males had higher incidences of chorionitis, anastomosis, unequal sharing of placenta, placental inflammation and placental lesions than females. Length of cords was slightly higher for males than females.

### Vascular anastomosis

Apart from the role of maternal nutrient restriction in reduction of fetal growth via placental amino acid transporter activity [2, 3], evidence suggests that sex specific adaptation of the placenta may be central to the differences in fetal growth and survival [11]. Studies consistently report that adverse fetal and neonatal morbidity relates to sex specific differences [12, 13]. However, sex specific differences of placental and cord pathology is rarely noted in the literature. Our study showed the importance of sex-specific findings related to the placenta and cord. Using the subject-specific approach, we found that males whose co-twin also was a male were at an increased risk for anastomosis compared with females from female-female twin pairs. Vascular anastomosis is characterized as an artery or vein captured by one twin where its partner artery or vein is captured by the second twin.

Differential vascular capture may result in discordant twin outcomes. A higher frequency of anastomosis in males compared with females was found in our analysis. Vascular anastomosis or sharing is almost foreseeable in MC twinning [14]. When we controlled for the effect of chorionicity, the adjusted odds for anastomosis was still 63% higher in males than females. Similarly, chorionicity adjustment for analysis of the relationship between anastomosis and sex pairing showed 70% higher odds of anastomosis in males from male-male twin sets compared with females from female-female twin sets. Moreover, the presence of a male in the uterus led to increased odds of vascular anastomosis in females who share the uterus with males (24% increase in odds, 95% CI = 0.54–2.83). This finding suggests that both single and double males in the uterus are associated with higher frequencies of anastomosis. This finding, however, should be interpreted carefully as we had a few cases of discordant gender among MC twins. These cases have been reported in case studies before and could be in the form of 46XY and 46XO (due to genetic mitotic disjunction) or cases of mosaicism [15]. It is also possible that these cases of discordant gender among MC twins are misclassified in the laboratory.

A MC twin placenta is designed to feed one fetus; hence, attempts to cater for the needs of twin fetuses are often suboptimal leading to growth discordance. DC placentas are designed to function separately, but vascular anastomoses are found in pathological examination. This could be due to a compensation mechanism adopted by the placenta for the lack of proper placental size to nourish competing twins. The twin fetal circulations are, therefore, rarely detached and several inter-twin vascular transportations of various kinds may be present, irrespective of chorionicity. In these circumstances, knowledge of fetal sex and sex pairing can assist clinicians to predict the adverse pathological findings and thus be prepared for clinical intervention or closer monitoring of twins. Our findings suggest that male fetuses deserve more attention, as higher frequencies of anastomosis are expected for them. Similarly, chorionitis, unequal placenta sharing, composite inflammation, and placental lesions are found with higher risk in gestations where at least one of the co-twin is male.

### Unequal placental sharing

Unequal sharing of the placenta is often found in MC placentas, which in turn results in growth discordance [16, 17]. Whether or not the unequal placenta sharing is more common in males because of genetic predisposition, or the discharged male hormones causes the unequal pattern of parenchymal sharing, remains unknown.

### Placental inflammation

In singleton pregnancies, intra-uterine infection/inflammation of male fetuses has been attributed to higher mortality of male newborns. A histological examination of placentas and umbilical cords of 446 infants born at 23 to 32 weeks, cultured for aerobic and anaerobic bacteria, showed that male infants were significantly more likely to have placental membrane bacterial infection than female infants [6]. Another prospective study of 437 singleton pregnancies investigated the histopathological placental findings [18]. Chronic placental inflammation was significantly more noted in male than female fetuses. The higher inflammation rate in males was attributed to immunological response by the mother that considers male fetus as a foreign body also known as transplant rejection [19]. We hypothesis that a similar mechanism is more likely to apply to twin pregnancies, as according to our results males of both sex concordant and sex discordant twin pairs had higher frequencies of inflammation compared with females of female-female twin pairs. No twin study to date has investigated the relationship between sex pairing and placental inflammation.

Due to inherited nature of the design, there is an intra-clinician variation in the pathological examination.

### Limitations and strengths

This study limited due to the use of retrospectively collected data. We had to search the old paper files to find pathology records for a year worth of data while the remaining files were available in pdf format on electronic system. Error during data entry is therefore possible.

The research presented here was made possible by a large population-based registry that contains information on approximately 99% of births in the province of BC. The full provincial data is comprehensive, reliable and uses standard software that identifies specific errors or omissions, if any, in the mother and baby records. The information in the registry is compiled from standardized forms completed by clinicians. The validity of the data is ensured by quality control measures including built-in warnings in the data entry software and periodic data screening [20].

We hand-searched pathology reports and extracted data from a hospital with the largest frequency of twin pregnancies during the same time (2000–2010). Access to the pathology records of placenta and cord from the hospital records enabled us to collect not only the chorionicity data but also assemble unique information such as use of selective reduction procedures, and the presence of papyrus placentas and vanishing twins.

To our knowledge, this research is unique in that it is the first to address specific micro- and macro-pathology findings in relation to sex discordance and placenta/cord adverse outcomes comprehensively. The number of studies in the literature related to placental pathological findings and cord length in twin gestations in relation to sex discordance is scarce.

The analytical strength of this research is employing the generalized estimating equations. Twins provide naturally matched pairs or clusters with within-twin-pair and between-pair effects. Specialized standard regression models are needed to reflect the paired structure of the data, which induces correlation between twins.

Given that the intra-uterine environment shared by a set of twins could impact the outcomes of interest in this study, we decided to utilize two analytical approaches to investigate the role of sex in relation to pathological outcomes. We first analyzed the data using a population-average approach where a selected twin from the population is compared with another twin who is also selected from the population. We then compared each twin with his or her mate, illustrating the use of a subject-specific-model rather than a population-based approach. Hence, sex pairing comparisons were adopted for analysis.

Based on the population average approach, we found that males had higher incidences of chorionitis, anastomosis, unequal sharing of placenta, placental inflammation and placental lesions than females. Length of cords was slightly higher for males than females. To the best of our knowledge, there is no study to date that replicates these findings in such a comprehensive manner.

## Conclusions

Ultrasound screening of twin gestation is mostly focused on the anatomical features of fetuses, on amniotic fluid volumes, and on umbilical vascular flow rather than sex. Clinicians look for complications such as TTTs, growth discordance, fetal death of one twin, and the differences between superficial and deep vascular connections, rather than sex. Fetal sex is an important factor in placental findings including anastomosis, unequal placenta sharing, placental lesions and placenta inflammation. These signs and symptoms should be thoroughly screened, especially if the fetus is male and is from a male-male pair. Although these findings are clinically important, they might not lead to change in practice until more evidence is provided by basic sciences as what are the mechanisms behind these results.

## Abbreviations

BC: British Columbia
BWD: Birth weight discordance
C&W: Children and Women’s
CI: Confidence interval
DC: Dichorionic
FF: Female-female
FM: Female-male
GEE: Generalized estimating eq.
MC: Monochorionic
MM: Male-male
PSBC: Perinatal Services British Columbia
TTTs: Twin to twin transfusion syndrome
